# Development of a Mucoadhesive Liquid Crystal System for the Administration of Rifampicin Applicable in Tuberculosis Therapy

**DOI:** 10.3390/life12081138

**Published:** 2022-07-28

**Authors:** Kaio Pini Santos, Camila Fernanda Rodero, Camila Maríngolo Ribeiro, Maria P. D. Gremião, Rosângela Gonçalves Peccinini, Fernando Rogerio Pavan, Camron Pearce, Mercedes Gonzalez-Juarrero, Marlus Chorilli

**Affiliations:** 1 Department of Drugs and Medicines, School of Pharmaceutical Sciences, São Paulo State University (UNESP), Araraquara 14800-903, Brazil; camilafrodero@hotmail.com (C.F.R.); palmira.gremiao@unesp.br (M.P.D.G.); marlus.chorilli@unesp.br (M.C.); 2 School of Pharmaceutical Sciences, São Paulo State University (UNESP), Araraquara 14800-903, Brazil; maringolocamila@gmail.com (C.M.R.); peccinin@fcfar.unesp.br (R.G.P.); fernando.pavan@unesp.br (F.R.P.); 3 Mycobacteria Research Laboratories, Department of Microbiology, Immunology & Pathology, Colorado State University, Fort Collins, CO 80523, USA; camronpearce@gmail.com (C.P.); mercedes.gonzalez-juarrero@colostate.edu (M.G.-J.)

**Keywords:** liquid crystal system, rifampicin, drug delivery, tuberculosis

## Abstract

Since 1966, rifampicin (RIF) has been considered one of the most potent drugs in the treatment of tuberculosis (TB), which is caused by infection with M. tuberculosis (Mtb). New nanostructured formulations for RIF delivery and alternative routes of administration have been studied as potential forms of treatment. This study evaluates a liquid crystal system for RIF delivery, using alternative drug delivery routes. The systems developed are composed of surfactant, oleylamine, and soy phosphatidylcholine. With the aid of polarized light microscopy, it was possible to determine that the developed systems had a hexagonal mesophase. All systems developed showed non-Newtonian pseudoplasticity and a high degree of thixotropy. Liquid crystal systems with RIF showed an increase in elastic potential, indicating greater mu-coadhesiveness. The evaluation of mucoadhesive forces revealed an increase in the mucoadhesive potential in the presence of mucus, indicating the presence of satisfactory mucoadhesive forces. The 9DR and 10DR liquid crystal systems, when submitted to Differential Scanning Calorimetry analysis, remained structured even at temperatures above 100 °C, showing excellent stability. The developed liquid crystal systems showed a tolerable degree of cytotoxicity and bactericidal potential, for example, the 9DR system demonstrated a reduction in bacterial load after the third day and reached zero CFU on the seventh day of the test. The developed systems were also evaluated in the preclinical model of Mtb-infected mice, using the nasal, sublingual, and cutaneous route for the delivery of RIF associated with a nanostructured liquid crystal system as a possible tool in the treatment of TB.

## 1. Introduction

*Mycobacterium tuberculosis* (Mtb) is an aerobic mycobacterium and the etiologic agent of the disease known as tuberculosis (TB). In 2019, TB was the leading cause of death from a single infectious agent, with an estimated 10 million people contracting the disease and 1.4 million people dying from complications regarding TB disease progression. One predictive model suggests that the COVID-19 pandemic has reversed any progress made toward a global reduction in TB in 2020, bringing the global TB deaths back to what they were in 2012 [1]. One of the most potent first-line drugs in the treatment of tuberculosis is rifampicin, with a 95% effectiveness in cases of TB caused by susceptible strains [2]. However, due to the lipophilic character of rifampicin, low bioavailability, pre-systemic metabolism, and possible drug interactions, patients often report side effects and may develop rifampicin-resistant TB [3,4]. New alternatives have been studied to improve RIF bioavailability. Lately, with the advent of nanotechnology, numerous different nanostructured vehicles have been developed with the objective of increasing selectivity and effectiveness to reduce the drug dose and unwanted side effects. Among the nanostructured systems, we highlight liquid crystal nanostructures as a drug delivery system [5,6,7]. Lyotropic liquid crystals are formed by surfactant molecules that have a polar region and an apolar region. Liquid crystals are described as structures on two or three-dimensional nanometric scales with hydrophilic and hydrophobic characteristics and organized in mesophases that can vary between hexagonal, lamellar, or cubic [8]. These systems are usually formed by spontaneous structural organization, thus, production is relatively simple and low cost [9]. Due to their porous and highly uniform nanometric structure, liquid crystals can exist in different phases, each with unique properties that can favor the solubilization of active particles in aqueous, lipophilic, or amphiphilic compartments. Additionally, liquid crystals can be organized to form a rigid, drug-carrying matrix that has the potential for controlled drug delivery [7]. The advent of systems such as liquid crystals has increased studies of other possible routes for the delivery of rifampicin for drug therapy, such as sublingual, nasal, and cutaneous administration [10]. Recent studies characterize liquid crystals and explore the possibility of their application through different routes for drug administration [11,12,13,14]. The naturally high viscosity of liquid crystals allows fixation of the formulation at a desired site of action after administration. Thus, the properties inherent to this system are candidates for the development of mucoadhesive systems for administration through little explored methods such as nasal, sublingual, and cutaneous routes [15]. Among the routes of administration tested in this work is intranasal and sublingual drug administration which offer a possibility of systemic and optimal drug exposure and are considered potential routes of administration for the treatment of pulmonary TB. In summary, the objective of this work was to develop mucoadhesive liquid crystal systems for the administration of rifampicin, enabling the use of alternative routes of administration, such as nasal, sublingual, and cutaneous routes.

## 2. Materials and Methods

### 2.1. Materials

Rifampicin, purity ≥ 97% (Sigma Aldrich, St. Louis, MO, USA); Heparin, 5000 U.I./mL, (BlauFarmacêutica^®^, Cotia, Brazil); Dopalen, ketamine 10% injectable, veterinary use (Ceva, Paulínia, Brazil); Calmiun, xylazine 2% injectable, veterinary use (Agener União Saúde Animal, São Paulo, Brazil); Bacter Bacterial agar (Becton Dickinson, East Rutherford, EUA); Cetylalcoholethoxylated 20 OE and propoxylated 5 OP-Procetyl AWS (Croda, Campinas, Brazil); Calcium chloride dihydrate (Merck, St. Louis, CO, USA); Potassium chloride (Merck); Sodium Chloride (Merck); soy phosphatidylcholine (Lipoid GMBH, Geneva, Switzerland); culture medium-Dulbecco’s Modified Eagle Medium (Hyclone, Logan, UT, USA); Brook Middlebrook culture medium-7H11 (Difcot, St. Louis, CO, USA); Oleylamine (Sigma Aldrich, St. Louis, CO, USA); Middlebrook-OADC (Sigma Aldrich, St. Louis, MO, USA); Neutral Red (Sigma Aldrich, St. Louis, MO, USA).

### 2.2. Liquid Crystal Development

Three different phase diagrams were developed, distinguishing themselves only by the oil phase. All the diagrams contained ethoxylated and propoxylated cetyl alcohol (Procetyl^®^ AWS) as a surfactant, deionized water for the aqueous phase, and the oil phase, soy phosphatidylcholine, oleylamine, and a mixture of oleylamine/soy phosphatidylcholine 1:1 (*m*/*m*), each named diagram D, diagram E, and diagram F, respectively. For each diagram, 36 different formulations were prepared, with proportions varying from 10 to 80% (*w*/*w*) of the components mentioned above. After adding all the components, the formulations were homogenized with Ultra Turrax (IKA-T25 basic) at 13,000 RPM, for 3 min and then kept at rest for 24 h [14].

### 2.3. Incorporation of Rifampicin in Liquid Crystal Systems

The incorporation of rifampicin occurred through the addition of the drug in a concentration of 0.15 µg·mL^−1^ solubilized in the oil phase of the systems; later, the other components were added and homogenized as previously described. This rifampicin concentration corresponds to 10 times the MIC value for Mtb strains [16].

### 2.4. Physico-Chemical Characterization of Liquid Crystal Systems

#### 2.4.1. Characterization of Liquid Crystals by Polarized Light Microscopy

The polarized light microscopy (MLP) characterization was performed using a polarized light microscope (Axioskop-Zeiss^®^). Samples for imaging were prepared by placing a drop of the LCS of interest on a slide and covering it with a cover slip. Images were taken using a 40× objective lens, allowing for the classification of isotropy and anisotropy, as well as sorting the systems for mesophase organization [17].

#### 2.4.2. Continuous Rheological Analysis

The reograms of the systems of liquid crystal were obtained using the AR2000ex rheometer (TA Instruments). The methodology used to study reograms was the plate/plate geometry, at 32 °C. Briefly, samples were applied to the bottom plate of the rheometer with the aid of a spatula to reduce shearing, followed by 3-min of rest to minimize the tension induced before the analysis. The shear rate used was 0.01 to 100 s^−1^ for the upward curve and 100 to 0.01 s^−1^ for the downward curve, both over a period of 120 s.

Flow calculations were analyzed using the Law of Powers model (Equation (1))

(1) $$\sigma =k\cdot \gamma^{n}$$

where k is the consistency index and n is the fluid behavior index; based on this mathematical model, a value of *n* greater than 1 characterizes a dilating fluid, a value of *n* less than 1 characterizes a pseudoplastic fluid, and *n* equaling 1 characterizes a Newtonian fluid [18].

#### 2.4.3. Oscillatory Rheological Analysis

The oscillatory analysis was performed using an AR2000ex rheometer (TA Instruments) using plate/plate geometry, according to the fluidity of each sample at a temperature of 32 °C. For the determination of the viscoelastic region of the samples, a range of shear stress from 0 to 50 Pa and frequency of 1 rad·s^−1^ was used. The oscillatory test was performed to determine the evolution of the elastic (G′) and viscous (G″) modules as a function of frequency (0.01 to 10 Hz) at an oscillatory amplitude voltage (1.00 Pa) [18].

#### 2.4.4. Assessment of Mucoadhesive Forces

Mucoadhesive forces were evaluated using a TA-XT plus texture analyzer to measure the forces required to detach samples in contact with a model membrane (porcine nasal mucosa). To perform the test, the probe was initially covered with porcine nasal mucosa; then, the mucosa-coated probe was moistened with simulated nasal fluid (SNF) consisting of 8% mucin (*w*/*v*), NaCl (7.45 mg·mL^−1^), KCl (1.29 mg·mL^−1^), and CaCl_2_. 2H_2_O (0.32 mg·mL^−1^) and the pH was adjusted to 6.0; then the analysis began. SNF was added to the LCS in the proportion of 1:1 (*m*/*m*) to simulate the conditions of the nasal mucosa (porcine mucosa) and evaluate the effects of the LCS phase change from the addition of SNF. Water was used as a negative control under the same conditions. During the analysis, the probe, covered with the porcine nasal mucosa, was inserted 1.5 mm deep into the sample for 60 s, without any work being applied during the phase of mucosa-sample contact, and then removed at a constant speed of 0.5 mm·s^−1^ until the probe was no longer in contact with the mucosa-sample. With the aid of the Texture Exponent Lite software, the force necessary to rupture the mucosa sample was verified using a graph of strength as a function of time to calculate the area on the curve obtained during the rupture phase, this value being designated as mucoadhesion work (maW). Another parameter determined in the analysis was the maximum force obtained during the removal of the probe, known as the mucoadhesion peak (maP) [13].

#### 2.4.5. X-ray Scattering at Low Angle (SAXS)

Low angle X-ray scattering measurements were taken on the SAXS line at the National Laboratory of Luz Sincrotron (LNLS), Campinas, SP. The SAXS 1 line, equipped with a set of asymmetric slits and a silicon monochromator, generated a monochrome beam of λ = 1608 Å and a cross-section of approximately 1.6 mm^2^. A position-sensitive X-ray detector and a multichannel analyzer were used to monitor the intensity of the scattered beam, I (q), as a function of the scattering vector module. The scattered intensity was normalized by the thickness and the attenuation of the sample and all measurements were performed under ambient temperature and pressure conditions [19].

#### 2.4.6. Differential Exploratory Calorimetry Analyses

The equipment used was a DSC microcalorimeter (DSC-TA Instruments, DSC-Q10). Differential exploratory calorimetry (DSC) analyses were performed in order to observe how the constituents of LCS behave with each other; in addition, these analyses allowed for the evaluation of the interaction of rifampicin with the other constituents used in the preparation of formulations. A “reference” test was performed from a water-water curve, in addition to DSC analyses with all selected formulations (with and without rifampicin). An analysis was also performed with free rifampicin, without the interference of the constituents of the liquid crystalline systems [20].

### 2.5. Cytotoxicity Tests (Agar Diffusion Method)

The L929 cell Line (fibroblast) was cultured at 37 °C in Dulbecco DMEM medium with 10% fetal bovine serum in an atmosphere of 5% CO_2_. Initially, 4 mL of a cell suspension at a concentration of 2 × 10^5^ mL^−1^ cells was pipetted into each well of 6-well plates (Costar^®^), followed by incubation at 37 °C with 5% CO_2_ for 48 h to allow adhesion and confluence of a cell monolayer. After 48 h, the culture medium was aspirated, and each well was washed with 2 mL of phosphate buffered saline pH 7.4 (PBS). After the PBS was aspirated, 1 mL of the covering medium, made with Dulbecco DMEM medium 2× (1:1 *v*/*v*) containing 1.8% agar and 0.01% neutral red dye, was added to each well. Filter paper discs were soaked in LCS and then placed in the center of the well containing agar. A filter paper disc soaked in DMEM culture medium was added to one well as a negative control, and for the positive control, a filter paper disc soaked in TRITON-X was added to a separate well. Plates were then wrapped in aluminum foil to avoid cell damage from the photoactivation of neutral red and incubated at 37 °C with 5% CO_2_ for 24 h. After incubation for 24 h, the wells were macroscopically observed for the formation of a clear halo around the samples and positive controls as evidence of effects related to cytotoxicity. This methodology provides quantitative data by measuring each halo in centimeters and qualitative information based on the interpretation of results [21]. Results were interpreted by correlating the final mean obtained for each sample to the degree of cytotoxicity as shown in Table 1.

### 2.6. Bacterial Killing Kinetics of the RIF Carrying LCS, a Bacterial Inoculum of Mtb

A H37Rv strain was exposed to the selected systems of liquid crystal and the reduction in the number of colony-forming units (CFU) was analyzed over time. The bacillus suspension was prepared from an inoculum on a McFarland scale equal to 1.0, diluted 20-fold to achieve a final mycobacterial concentration near 10^5^ CFU mL^−1^. The RIF solution was prepared with dimethyl sulfoxide (DMSO) at a concentration of 10 mg·mL^−1^. The concentration of RIF carried by the LCS was 1.5 μg·mL^−1^, 100 times higher than the reported MIC for RIF against Mtb H37rv (0.015 μg·mL^−1^) [17]. Mycobacterial suspensions with systems of liquid crystal were incubated at 37 °C in a shaker, and aliquots were collected every 48 h over a period of 15 days. Each aliquot underwent decimal serial dilution, then 100 μL of each dilution was dispensed on plates containing 7H10 agar supplemented with OADC (solid medium for CFU counting). Agar plates were then incubated for up to 30 days at 37 °C, with 5% CO_2_, until colonies were visible to the naked eye for counting. The results were graphed on a curve representing the number of viable bacteria as Log_10_ CFU·mL^−1^ as a function of the exposure time in days.

### 2.7. In Vivo Assay

#### Mouse Model Infection with Mtb

All animal studies were performed at Colorado State University under certified Biosafety level III animal protocols and in accordance with the guidelines of the Colorado State University Institutional Animal Care and Use Committee. A total of 20 model female Balb/c mice, weighing between 20 to 25 g each, were used, with 5 animals per group (*n* = 5). The animals were infected using an established low-aerosol protocol calibrated to deliver 50 a 100 colony forming units (CFU) into the lungs of mice.

Mice were exposed to a low-dose aerosol infection with Mtb using a Glass-Col inhalation exposure system. The virulent Mtb strain Erdman (TMCC 107) was used to infect mice. The Glass-Col inhalation system uses compressed air that is pumped into the chamber for 20 min through a venture nebulizer containing 5 mL of a bacteria suspension at a concentration of 2 × 10^5^ bacilli/mL. On day one post-infection, three mice were sacrificed to verify the bacterial deposition of 50 to 100 CFU per mouse. The bacterial burden in the lungs was monitored by plating serial dilutions of individual whole-organ homogenates onto nutrient 7H11 agar and incubation at 37 °C until CFU appeared on the agar plates (3–4 weeks later). The treatment was initiated on day 30 post-aerosol infection and continued for 4 weeks. The groups of mice (*n* = 5) were sacrificed on day 30 of treatment, and thereafter, the lungs and spleen were prepared for determination of bacterial burden. After 4 weeks of treatment, the mice were sacrificed in a CO_2_ chamber followed by cervical dislocation. At necropsy, the left lung and spleen of the mouse were collected and fully homogenized with the aid of a bullet blender. The plate was placed in a 37 °C incubator for 3–4 weeks after which CFU were counted. Bacterial burden in each sample was expressed as log_10_ CFU/organ. The LCS tested was the 9DR system; this system was evaluated for different administration routes (nasal, sublingual, and cutaneous). Treatment was started 30 days after the animals were infected. In the nasal group, 3 µL of the liquid crystal system was administered. For the sublingual group, 10 µL of the liquid crystal system was administered below the tongue of the animal in the sublingual portion, and in the cutaneous group, the animal’s abdominal hairs were removed using a hair clipper that did not cause pain or discomfort to the animals, then 100 µL of liquid crystal was massaged in this region. All groups underwent treatment five times a week for a period of 30 days.

## 3. Results

### 3.1. Liquid Crystal Development

**Figure 1:**
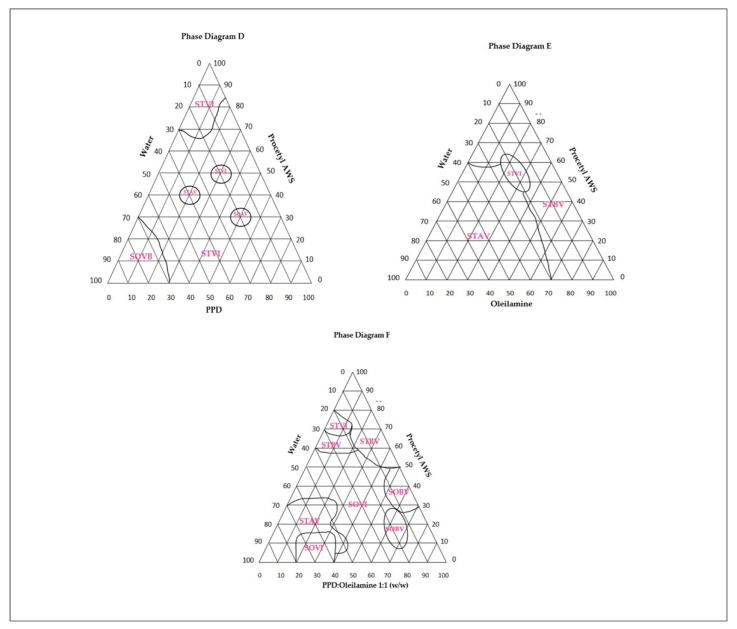
Diagrams of ternary phases were developed, D, E, and a pseudo-ternary F. The phase changes were visually identified and classified as: translucent intermediate viscosity systems (STVI), translucent high viscosity systems (STVA), translucent low viscosity systems (STBV), opaque intermediate viscosity systems (SOVI), opaque systems of high viscosity (SOAV), opaque low viscosity (SOBV), and phase separation (SF) systems.

Initially, two diagrams of ternary phases were developed, D and E, as well as one pseudo-ternary diagram, F. The difference between each of the phase diagrams was the constitution of the oily phase. Ternary diagram D had soybean phosphatidylcholine as the oil phase; ternary diagram E, oleylamine, and ternary diagram F had the 1:1 (*w*/*w*) mixture of soy phosphatidylcholine/oleylamine as the oil phase. Each diagram generated 36 formulations, with each classified according to its visual characteristics of transparency or opacity and viscosity. This classification gave rise to regions of different viscosities and transparencies within the diagram, as defined in Figure 1.

The selection of LCSs was carried out considering high fluidity, low viscosity, and thermodynamic stability. In each of the three unique diagrams, there were regions that satisfied these requirements for the use of biomucoadhesive LCSs and two systems were selected from each of these regions. Based on this, six different LCSs (two from each diagram) were selected for the continuation of the study, their composition is detailed in Table 2.

### 3.2. Incorporation of Rifampicin in Liquid Crystal Systems

The systems selected for rifampicin incorporation were 9D, 10D, 9E, 10E, 9F, and 10F, with a concentration of RIF in each precursor LCS of 0.150 µg mL^−1^. After 24 h of drug incorporation, no precipitate was observed, supporting the hypothesis that the entire drug was incorporated. The composition of each system is detailed in Table 3.

### 3.3. Physico-Chemical Characterization of Liquid Crystal Systems

#### 3.3.1. Characterization of Liquid Crystals by Polarized Light Microscopy

**Figure 2:**
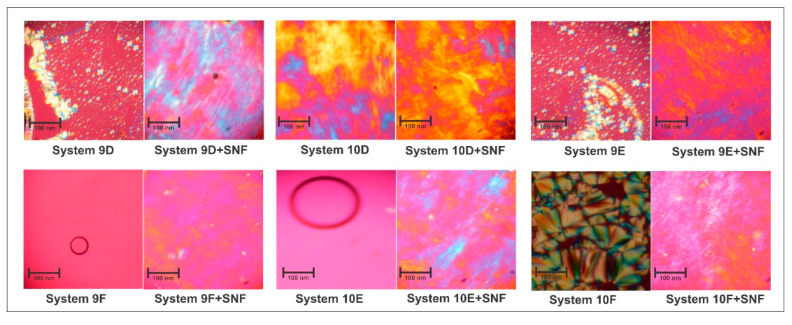
Photomicrographs of 9D, 10D, 9E, 10E, 9F, and 10F with and without the addition of SNF.

Photomicrographs of LCSs 9D, 10D, 9E, and 10F showed that the visualized systems correspond to LCSs, as it was possible to visualize specific structures such as Malta crosses and striations. In the 10E and 9F systems, the presence of precursor systems for liquid crystals was confirmed since, when observing the isolated system in the MLP, only dark fields were visualized. When adding SNF, all systems underwent a reorganization in their mesophase as shown in Figure 2.

#### 3.3.2. Continuous Rheological Analysis

Rheology is widely used in the characterization of drug-carrying systems, verifying the flow speed and spreadability of the systems. This technique is important for liquid crystal precursor systems since flow and spreadability are important parameters when it comes to intranasal administration [22]. With this in mind, the liquid crystal precursor systems 9DR, 10DR, 9ER, 10ER, 9FR, and 10FR, were selected for continuous rheological flow characterization and further evaluation of how each material behaves when exposed to stress [23].

**Figure 3:**
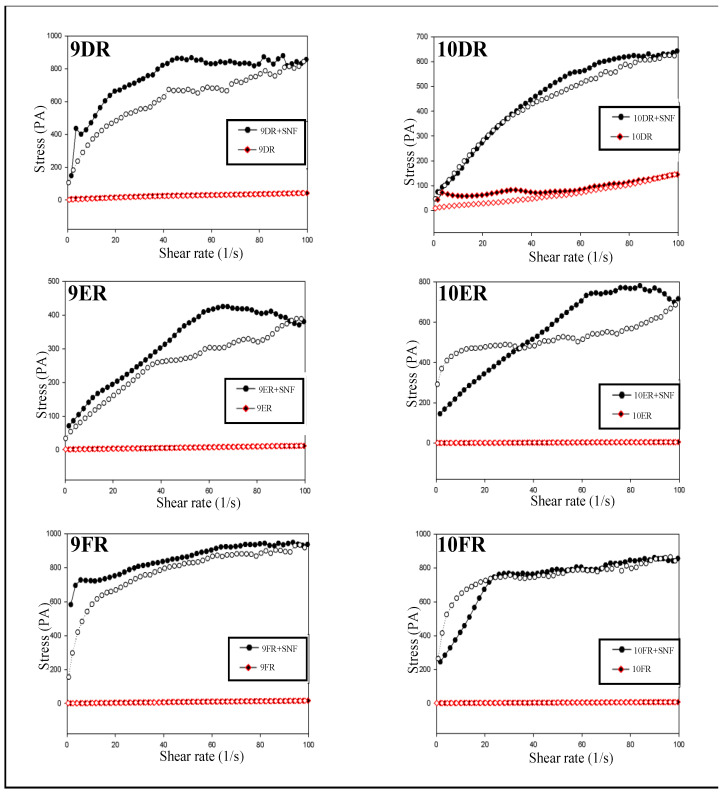
Reograms of the 9DR, 10DR, 9ER, 10ER, 9FR, and 10FR systems (upward/downward curve; in the presence and absence of SNF.

All of the selected systems presented pseudoplasticity due to their organized mesophase which causes greater resistance to flow, and, with the exception of the 10ER system, all systems demonstrated a high degree of thixotropy [14]. Thus, systems with a higher degree of thixotropy also have a higher degree of initial organization, which is directly proportional to the area of hysteresis in the flow curve [24]. The selected systems were also analyzed in the presence of SNF to characterize any change in flow and mesophase organization. Analyzing the flow curves of the systems with the incorporation of rifampicin, it was possible to conclude that the incorporation of rifampicin did not alter the behavior of the systems analyzed. All systems showed non-Newtonian pseudoplastic behavior. Regarding the analysis of the descending curve, there was no overlap of the descending curves for the 9DR, 9ER, 10ER, and 9FR systems, forming a history area (time-dependent thixotropic classification). The 10DR and 10FR systems recovered in a short period of time after the shear rate ceased were classified with thixotropic independent time, as shown in Figure 3.

In this model, *n* greater than 1 represents a dilating fluid, *n* less than 1 represents a pseudoplastic fluid, and *n* equal to 1 represents a Newtonian fluid. The degree of “pseudoplasticity” can be measured by the flow behavior (*n*), which increases with the decrease in pseudoplasty [25]. The viscosity of the formulations can be evaluated with the consistency index (*k*), which increases with the viscosity of LCS. The values of *n* and *k* are shown in Table 4. When comparing the analysis of systems containing rifampicin with SNF and without SNF, it was observed that as the amount of SNF in the systems is increased, the degree of pseudoplasticity becomes greater. All systems with the addition of SNF started to show a non-Newtonian pseudoplastic behavior.

#### 3.3.3. Oscillatory Rheological Analysis

**Figure 4:**
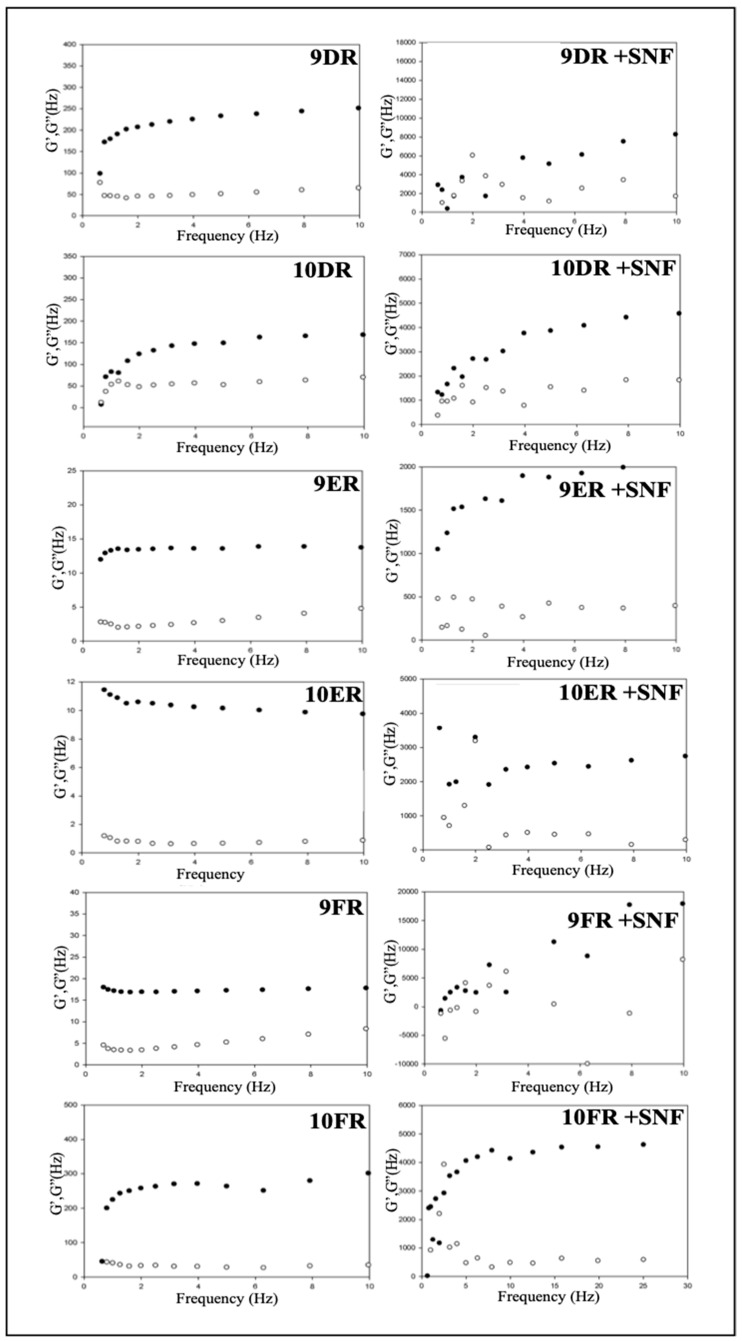
Reograms obtained for the 9DR, 10DR, 9ER, 10ER, 9FR, and 10FR systems in the absence and presence of SNF, the white circle represents G′ and the black circle represents G′′.

This assay provides information about the viscoelasticity of the sample, from the relationship of a sine wave and the shear stress, by generating a deformation in the sample and showing their viscoelastic characteristics, as well as defining the structural nature of the system which is directly related to its drug carrying performance. All systems containing rifampicin presented G′ greater than G″, as shown in Figure 4, indicating the predominance of elastic characteristics. The LCSs demonstrated greater structural organization with the addition of SNF which considerably increased the value of G′ by more than 100 times its original value while still maintaining the elastic characteristics that are directly related to a desirable mucoadhesiveness potential. Each system also exhibited a constant G′ value independent of the frequency used, a feature of more structured systems.

To quantify G′ dependence, an R-value was determined from a linear regression of the data obtained. Additionally, the viscoelastic component, *n*, was calculated through Equation (2), allowing for a more accurate assessment of the organizational structure of each system’s mesophase [26].

G′ = S·ω*ⁿ* (2)

where: G′ is the storage module, ω is the oscillatory frequency, S is the formulation resistance, and *n* is the viscoelastic exponent.

The parameters S and *n* are indicative of the cross-linking density inside the sample; the higher the value of S, the more cross-linked and organized the sample. The value of *n* is related to the increase in crosslinking, which can indicate formulations with a weaker structure. The values of r, S, and N are listed in Table 5.

#### 3.3.4. Assessment of Mucoadhesive Forces

**Figure 5:**
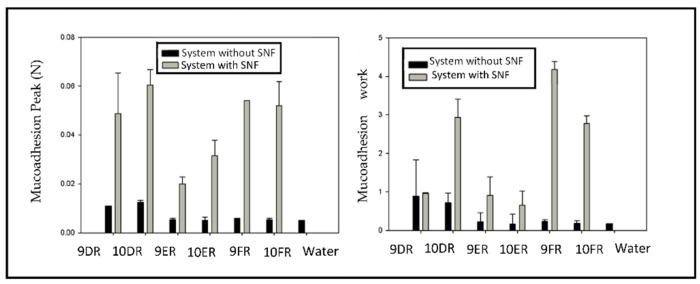
Graph illustrating the peak mucoadhesion (P_M_) and mucoadhesion work (W_M_) values of the 9DR, 10DR, 9ER, 10ER, 9FR, and 10FR systems with and without SNF.

The assessment of mucoadhesive forces provided information on the interactions occurring at the mucosa-system interface. Figure 5 shows peak mucoadhesion and the mucoadhesion work values, with and without SNF, for systems 9DR, 10DR, 9ER, 10ER, 9FR, 10FR, and water.

Analysis revealed that all systems had an increase in mucoadhesion force in the presence of SNF. System 9FR showed the greatest increase in mucoadhesion work, with nearly four times the value than without SNF.

#### 3.3.5. X-ray Scattering at Low Angle (SAXS)

**Figure 6:**
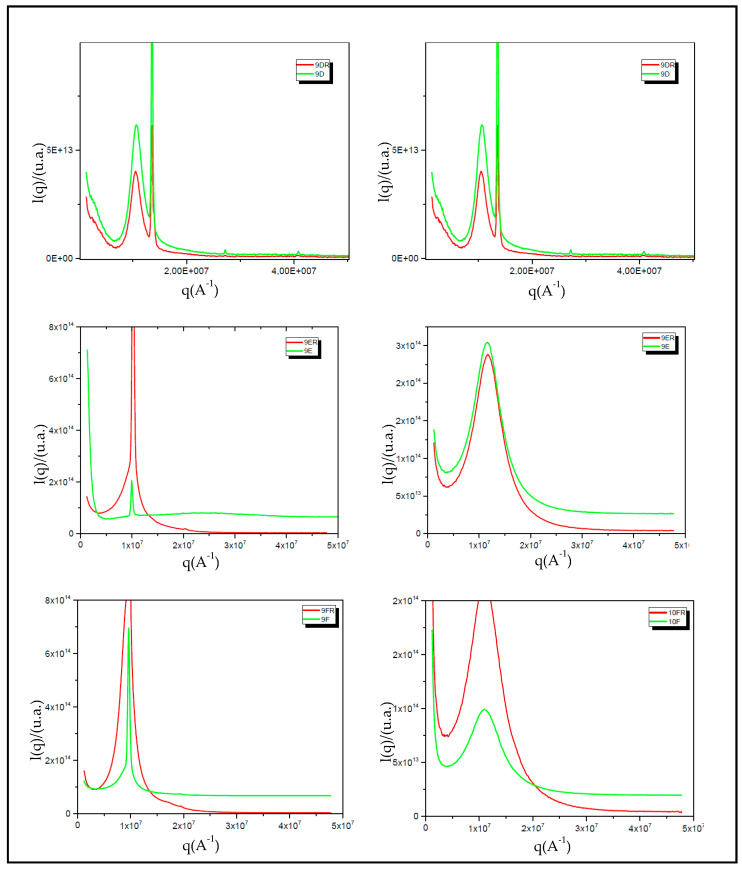
Curves of scattering intensities as a function of the scattering vector q.

The hexagonal structures of the liquid crystal can be checked according to the position of the diffraction peaks in the axis of the scattering vector, q, as shown in Figure 6. For lamellar structures, the relative position of the peaks (in relation to the first most intense peak) obeyed the 1 ÷ 2 ÷ 3 ratio, while for hexagonal structures (Figure 1), the ratio was 1 ÷ 3 ÷ 2 ÷ 7. Therefore, the results obtained by the SAXS analysis corroborate the results of polarized light microscopy. These results provided more accurate results on the nanostructure of the 9D, 10D, 9E, 10E, 9F, and 10F systems and of these rifampicin-bearing systems (9DR, 10DR, 9ER, 10ER, 9FR, and 10FR). The qmax scattering andlamellar and hexagonal periodicity values of liquid-crystalline formulations are described in Table 6.

#### 3.3.6. Differential Exploratory Calorimetry Analyses (DSC)

**Figure 7:**
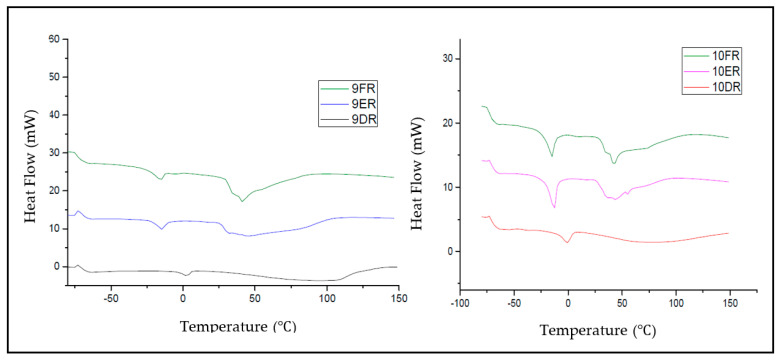
DSC curves of the 9FR, 9ER, 9DR, 10FR, 10ER, and 10DR systems.

DSC is a thermoanalytical technique that measures the difference in the amount of heat needed to increase the temperature of a sample as a function of time. To determine the amount of free and bound water in each system, DSC was performed for the 9DR, 10DR, 9ER, 10ER, 9FR, and 10FR systems as shown in Figure 7. The DSC analyses also showed that the systems 9ER, 9FR, 9ER, and 10FR have a lower heat capacity compared to 9DR and 10DR systems. The 9ER, 9FR, 10ER, and 10FR systems appear to undergo a disruption in their crystalline network when subjected to temperatures above 50 °C. The 9DR and 10DR systems, on the other hand, remain structured at temperatures above 100 °C.

### 3.4. Cytoxicity Tests (Agar Diffusion Method)

Prior to in vitro testing, cells were cultured to confluency and tested for ~90% viability. Next, the cytotoxic level of the liquid crystalline systems was qualitatively analyzed. The cytotoxicity of each system was evaluated by measuring the size of the formed halo around a filter paper disc embedded in the system. Cytotoxicity presented as null, mild, moderate, or high according to the size of the halo, as shown in Table 1. The negative controls (culture medium) did not present a halo formation and were classified as grade 0 cytotoxicity (null). The positive controls (Triton-X) had a halo formation greater than 1.0 cm, which is indicative of severe cytotoxicity. All systems, apart from the 9DR system which was classified as having light cytotoxicity, presented moderate cytotoxicity, as presented in Table 7.

### 3.5. Bacterial Kinetics

Bacterial kinetic assays were performed in triplicate to evaluate the bactericidal profile of liquid-crystalline systems 9D and 10D (systems without rifampicin) and 9DR and 10DR systems (with rifampicin). Rifampicin was added to the liquid crystalline systems at 1.50 μg·mL^−1^, which is 100 times the accepted MIC value of rifampicin against the H37rv strain (0.015 μg·mL^−1^). In this assay, the bacterial inoculum is exposed to either free LZD or LZD carried by delivery systems, and the reduction in the number of colony-forming units (CFU) was assessed over time.

**Figure 8:**
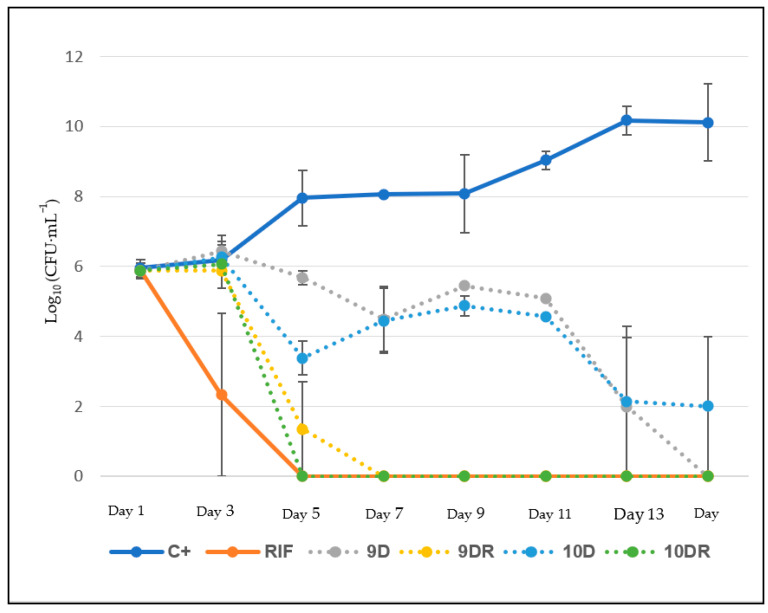
Graphic representing the number of viable bacteria in Log_10_ (UFC·mL^−1^) as a function of the exposure time in days.

The bacilli suspension was prepared from an inoculum on a McFarland scale equal to 1.0 and diluted 20 times for an initial concentration of bacteria approximated to 10^5^ CFU·mL^−1^. Figure 8 shows any changes in Log10 (CFU·mL^−1^) throughout the bacterial kinetics assay for each of the selected liquid crystalline systems and respective controls. The liquid crystalline systems demonstrated bactericidal activity by a reduction in CFU after the third day of the assay. System 9D, containing rifampicin (9DR), demonstrated a reduction in bacterial load after the third day and reached zero CFU by the seventh day of testing. However, for the 10D crystalline liquid system with rifampicin (10DR), this same effect was observed from the fifth day of the test.

### 3.6. In Vivo Assay

#### Mouse Model Infection with *M. tuberculosis*

**Figure 9:**
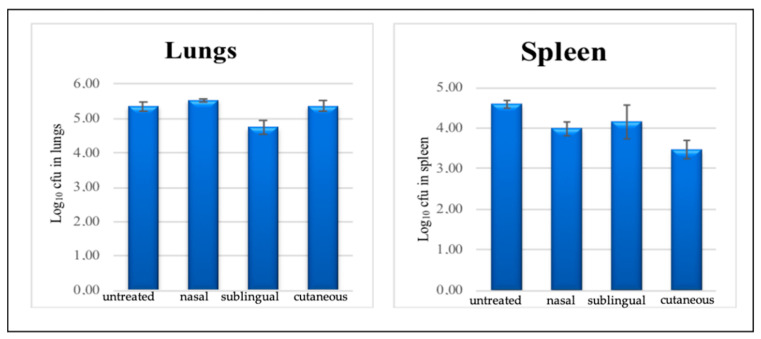
Evaluation of liquid crystal systems (9DR) in BALB/c mice following low-dose aerosol infection with *M. tuberculosis*. The bars represent the mean log_10_ CFU value from the spleen of individual BALB/c mice. Mice were treated for 4 weeks with systems 9DR through three different administration routes (group 0 = untreated/group, 1 = nasal route/group, 2 = sublingual route, and group 3 cutaneous route).

The in vivo efficacy of LCS 9DR against Mtb was evaluated using the chronically infected BALB/c mouse model. The comparative analysis of total CFU in the lungs between different groups of mice receiving LCS 9DR by different administration routes suggested the efficacy of treatment when RIF was administered by the sublingual route. However, the comparative analysis of the log10 CFU obtained after plating the spleen homogenates from each mouse using different administration routes demonstrated no significant differences between groups of mice as shown in Figure 9.

## 4. Discussion

The ternary phase diagram D obtained by mixing Procetyl^®^ AWS, soy phosphatidylcholine, and water is shown in Figure 1. Nearly all of the systems obtained from this diagram have a hexagonal mesophase where the surfactant concentration is greater than 70% and less than 20% water, except for a small portion of the upper vertex. This probably occurs due to an agglomeration in the molecules of the surfactant, where the polar heads turn to the internal aqueous medium, obtaining a minimum of free energy, forming reverse micelles, very common in the lamellar phase [17]. In the left vertex of phase diagram D, a dark field region was evidenced with systems classified as SOBV; perhaps because the higher water content destabilized these systems, generating isotropic systems.

**Figure 10:**
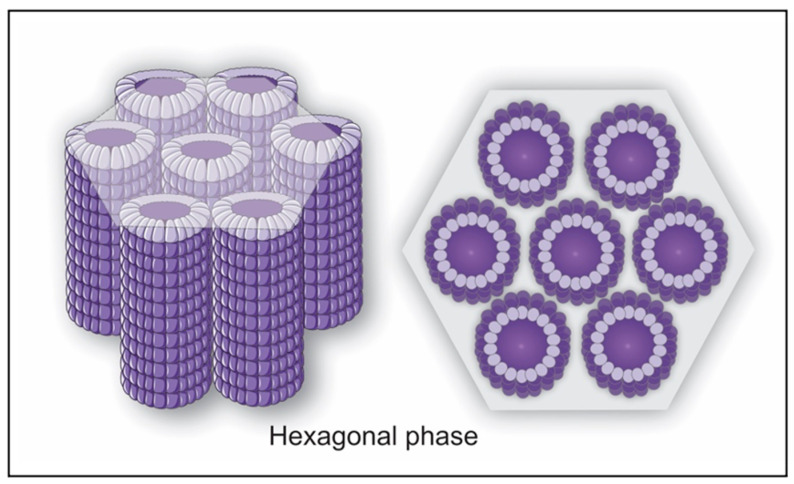
Schematic illustration of the hexagonal phase.

The ternary phase diagram E, obtained by the mixture of Procetyl^®^ AWS, soy phosphatidylcholine/oleylamine 1:1 (*w*/*w*), and water, is shown in Figure 1. Relative to diagram D, the phase diagram E presented systems of high fluidity and low viscosity, probably due to the high fluidity of oleylamine (oily phase). This diagram shows the predominance of a dark field in the upper right vertex, a region where the systems present less than 30% water. The water and oleylamine are stabilized by the presence of a film formed by Procetyl^®^ AWS (located at the interface between water and oleylamine), which results in high stability and transparent systems that are characteristic of microemulsions [27]. A higher level of organization was observed in the mesophase of systems with greater than 30% water content. In these systems, surfactant molecules rearrange themselves into an elongated, highly organizational level, a characteristic event in the hexagonal mesophase (Figure 10). We suggest that water is dictating the level of structural organization in this diagram, where systems with less than 30% water have microemulsion characteristics, systems with more than 30% water have characteristics of a hexagonal mesophase, and systems in the left vertex, with more than 70% water, presented high viscosity and zero fluidity, which is characteristic of a cubic mesophase and high molecular organizational index.

Finally, the ternary phase diagram F, composed of a mixture of Procetyl^®^ AWS, phosphatidylcholine/oleylamine 1:1 (*w*/*w*), and water, is shown in Figure 1. The upper vertex of diagram F is characterized as a dark field in the MLP and contains low viscosity and high fluidity systems which are characteristic of an isotropic system. Regions with 20% water also presented a dark field, but within these regions, there were major differences in the characteristics of systems. Between 10% and 20% of the oil phase, systems are called STBV (translucent systems of low viscosity) and meet the characteristics of microemulsion because of their high surfactant rates (60%), decreased droplet size, and optical transparency [27]. Hexagonal mesophases were observed in the medial region of the diagram. This was possibly due to the presence of phosphatidylcholine which can form a hexagonal mesophase at a water concentration between 20% and 70%. It should be noted that in diagram E, where the oil phase is composed only of oleylamine, the formation of hexagonal mesophase also occurred in this same medial region. These observations reinforce the hypothesis that the two oily components have synergistic effects in the formation of the hexagonal mesophase.

All three phase diagrams (D, E, and F) presented systems that met the requirements for administration outside of conventional (oral) routes, and two of each diagram were selected with characteristics of high fluidity, low viscosity, and stability. From this, the six different systems, shown in Table 2, were selected for further analysis and application.

System 9D suffered a rearrangement in its mesophase from lamellar to hexagonal with the addition of SNF. In the lamellar phase, the polarized light plane of the microscope was rotated when moving in a direction perpendicular to the plane of the ordered phase, displaying characteristics of the Malta cross [28]. After adding SNF to system 9D, it was found that the plane of polarized light was rotated in all axes except the long axis, which generated a fibrous and striated optical structure characteristic of the hexagonal mesophase [25], shown in the MLP images of Figure 2. System 10D, on the other hand, was classified as STVI and presented streaks consistent with a hexagonal phase. With the addition of SNF to the system, 10D became highly viscous and had zero fluidity, yet still maintained its hexagonal mesophase. MLP imaging revealed clear and striated structural formation, indicative of an increase in the degree of structural mesophase organization. Similar to system 9D, the addition of SNF to system 9E resulted in a transition from a lamellar mesophase to a hexagonal mesophase. Initially, the Malta cross structures were observed, referring to the lamellar mesophase, but after the addition of SNF, the previously defined characteristics of a hexagonal mesophase were observed.

System 9F was characterized as a microemulsion through the verification of a dark field. After the addition of SNF, poorly defined structures with streaks, reminiscent of stretch marks, could be observed, but still with great similarity to the dark field. This was due to the transition from the original system to the hexagonal phase, and later to a possible cubic phase. In system 10F, the Malta cross is again observed, referring to the lamellar mesophase. As noted earlier with system 9F, the addition of the SNF to 10F induced a structure with little definition and vague streaks, but still maintained a similarity to the dark field. We suggest this is a mesophase in transition from the hexagonal phase to the cubic phase [29]. Among the numerous possibilities of mesophase that liquid crystals offer, in this work, we sought a formulation that initially presented a low viscosity mesophase (with a low level of organization), such as the lamellar or hexagonal mesophase, because after coming into contact with the mucus, it assumes a greater degree of organization in its mesophase, such as hexagonal mesophase with a greater degree of organization or cubic mesophase, thus increasing its mucoadhesive potential. Following the MLP characterization of each of the six LCSs, RIF was added at 0.150 µg·mL^−1^ and six new systems were generated (9DR, 10DR, 9ER, 10ER, 9FR, and 10FR), as shown in Table 2. No precipitate was observed 24 h after the incorporation of the RIF, reinforcing the hypothesis that all the drug was incorporated into the transporter; when rifampicin is transported by a liquid crystalline system, rifampicin is protected in the mesophase of the nanostructured system. Theoretically, this trap prevents drug degradation, increasing its stability in relation to free rifampicin [9].

The continuous rheology analysis showed that all systems with rifampicin display non-Newtonian pseudoplastic behavior. Analysis of the descending curve revealed an overlap of the descending curves for the 9DR, 10DR, 9ER 9FR, and 10FR systems, which formed a hysteresis area (rheopetic classification). The 9F, 10F, 9DR, 10DR, 9ER, 10ER, 9FR, and 10FR are pseudoplastic systems due to their organized mesophase which causes greater resistance to flow [17]. Apart from the 10ER system, these same systems all demonstrated a high degree of thixotropy, resulting from a mesophase of a higher level of organization that was only destabilized with high shear rates. Thus, these systems with a higher degree of thixotropy also had a higher degree of initial structure. The larger the area of hysteresis in the flow curve, the greater the microstructurization of the liquid-crystalline networks of the system [18]. All systems, after the addition of SNF, began to show a non-Newtonian pseudoplastic behavior, characterized by the non-constant viscosity that was directly affected by a change in the shear rate. Additionally, the pseudoplastic characteristics presented by all systems with the addition of SNF indicate that the systems tend to decrease in viscosity when the shear rate increases [26]. The rheological results demonstrate the feasibility of using these systems for the routes proposed in the work. During administration, high shear rates will decrease the viscosity of the systems, facilitating their spreadability and applicability; decreasing shear rates then leads to an increase in viscosity, which facilitates adherence and makes it difficult to eliminate rifampicin through mucociliary clearance mechanisms. It was observed that all systems presented a high degree of thixotropy after the addition of SNF. Thixotropy is directly related to an interaction between the components that constitute the system. Such interactions can only be changed by increasing shear speed and are easily recovered when the shear speed decreases. Thus, systems with a higher degree of thixotropy also have a higher degree of initial structure. The increase in the level of organization for each system is represented by the hysteresis area shown in the graphs in Figure 2. This increase in thixotropy in the presence of mucus is extremely important for mucoadhesive systems intended for nasal administration [18].

The oscillatory rheology analysis revealed that all systems exhibited G′ greater than G″, a characterization of systems of higher structure. The addition of SNF further increased the structural organization of the system, with the average G′ value increasing by 100 times its original value. These results corroborate with the results from the polarized light microscopy analysis, where the LCSs, after the addition of SNF, began to exhibit streaks under the polarized light, suggesting the presence of a hexagonal mesophase with a higher level of organization, and acquiring a higher viscosity and mucoadhesive characteristics. Furthermore, the LCSs with SNF exhibited larger elastic modules, indicating that mucus contact can promote the formation of a liquid-crystalline matrix with elasticity superior to the initial system, a property that can contribute to increased mucoadhesion of a drug delivery system [30].

The assessment of mucoadhesive forces revealed that all systems had an increase in mucoadhesion forces with the incorporation of RIF. The system with the greatest increase was the 9FR, increasing the value of mucoadhesive forces by approximately four times. The 9ER system obtained a W_M_ value of 0.907 N·s. This same system without rifampicin has a W_M_ value of 0.172 N·s, showing an approximate five time increase. The increase in mucoadhesive forces in systems with RIF is likely related to the molecular structure of this drug, which contains amine groups capable of undergoing protonation at acidic and neutral pH. This ionization guarantees an increase in electrostatic interactions at the mucosa-system interface, and through interactions with charged groups of the glycoproteins in the mucus, confers a considerable increase in mucoadhesive forces [31].

SAXS X-ray scattering analysis was performed to confirm the results from polarized light microscopy and provide more quantitative data on the nanostructure of the 9D, 10D, 9E, 10E, 9F, and 10F systems with and without RIF (9DR, 10DR, 9ER, 10ER, 9FR and 10FR). Figure 6 shows the scattering intensity curves as a function of the scattering vector q. The hexagonal, lamellar, and cubic structures of lyotropic liquid crystals were verified according to the position of the diffraction peaks on the axis of the scattering vector q. For lamellar structures, the relative position of the peaks (in relation to the first most intense peak) generally follows the 1 ÷ 2 ÷ 3 ratio, while for hexagonal structures, the expected ratio is 1 ÷ 3 ÷ 2 ÷ 7 [32]. For the cubic phase liquid crystals, the values correlated are 1.41 ÷ 1.73 ÷ 2.82 ÷ 3 [33]. From the graphs selected in Figure 6 and values assigned in Table 4, it was possible to identify which of the selected systems presented a mesophase crystalline structure. The results obtained by the SAXS analysis support the results from the polarized light microscopy analysis.

Through the analysis of the DSC, it was possible to determine the percentage of free water in each system and verify that, with RIF incorporation, the systems 9DR, 10DR, 10ER, 9FR, and 10FR suffered a decrease in the percentage of free water in the intermolecular spaces. This data suggests that the addition of RIF into the crystalline network provided a greater structure of water; due to the presence of the drug, the link sites increased, favoring greater structuring of the system. The DSC analysis also showed that the 9ER, 9FR, 9ER, and 10FR systems have a lower heat capacity when compared to the 9DR and 10DR systems, as shown in Figure 7. Systems 9ER, 9FR, 9ER, and 10FR appear to undergo degradation in their crystalline network at temperatures greater than 50 °C, whereas 9DR and 10DR remained structured at temperatures greater than 100 °C. This event may be attributed to the oleylamine in the 9ER, 9FR, 9ER, and 10FR systems since its boiling point is around 350 °C. Once again, these results corroborate the results of rheology, demonstrating the highest stability in systems consisting of phosphatidylcholine.

The cytotoxicity assays revealed that, apart from the 9DR system which was classified as mild cytotoxicity, all of the systems presented moderate cytotoxicity. All of the selected systems, both with rifampicin and without rifampicin, contained Procetyl^®^ as their main surfactant. It is well known that surfactants, in general, have a cytotoxic character because they cause structural disorganization in the cell membrane, causing cell lysis [33]. However, these cytotoxicity results suggest that the surfactant concentration probably favored the structuring of the liquid-crystalline system. The nonpolar region of the surfactant is likely binding to nonpolar sites of rifampicin (and other constituents of the oil phase) which causes steric protection of binding sites that would, under normal conditions, bind and destabilize cell membrane proteins. This would account for the decreased cytotoxic character of the system and guarantee the sustained release of rifampicin [34].

All the RIF-containing liquid crystalline systems presented bactericidal activity, as demonstrated by the drop in the CFU from the third day of release in Figure 8. This lag period is the time necessary for mesophase reorganization and to begin relaxing the crystalline network for the gradual release of RIF. The 9DR liquid crystalline system demonstrated a decrease in bacterial load from the third day onwards, reaching zero detectable bacteria by the seventh day. However, for the 10DR liquid crystalline system, this same effect was observed on the fifth day of the test. The difference in bactericidal effects may be because of the differences in intermolecular bonds that RIF makes with the constituents of the system. For example, system 10DR has a higher concentration of phosphatidylcholine and a lower concentration of water. Additionally, phosphatidylcholine has a high affinity for membrane phospholipids which results in an imbalance in the Mtb cell envelope [34], possibly accelerating RIF entry into the bacteria. Another important factor that influences the rate of drug release is the structural organization of the system. The 9DR system has a lamellar-type mesophase, and when in contact with the culture medium (aqueous solution), the system reorganized and acquired a hexagonal mesophase of a higher organizational level. However, it has been described that liquid crystalline systems with a hexagonal mesophase favor a slower drug release [34,35], an effect reflected in bacterial kinetics until the fifth day of the experiment. Subsequently, a total dilution of the system probably occurred, releasing the drug at a higher speed.

The liquid crystalline system 10DR has hexagonal mesophase, with a high level of structural organization, and after the third day of the experiment, it enters directly into a transition phase between hexagonal mesophase and total system disruption. By the fifth day of the experiment, the system is completely unstructured and all of the RIF is released into the culture medium, represented by a greater amount of free rifampicin and a significant decrease in CFU. Interestingly, even without RIF, systems 9D and 10D showed a small decrease in CFU after day 11 of the experiment. This effect was probably due to the presence of phosphatidylcholine, guaranteeing interaction with the Mtb cell envelope since the lipid wall is made up of phospholipid similar to phosphatidylcholine. The presence of this destabilizes the membrane and may ultimately lead to its rupture and cell death [36].

This primary test leads us to suggest that there is a synergistic effect between the mesophase reorganization of the liquid-crystalline systems releasing the drug and the presence of phosphatidylcholine disrupting the microorganism’s cell membrane, since these mechanisms happen simultaneously, generating a decrease in bacterial load. However, it is important to emphasize that these hypotheses still need to be verified with other tests.

In in vivo studies, it was possible to observe that the intrinsic characteristics of the liquid crystalline system seem to favor mucoadhesion [31], allowing the system to remain in the nasal mucosa for a longer time, possibly favoring a slower permeation of the RIF [32]. A comparative analysis of the total bacterial load in the lungs, total CFU in the lungs (Figure 9), among mice that received 9DR LCSs by different routes of administration, suggested a therapeutic action. We speculate that both the sublingual and nasal regions are highly vascularized, allowing for the rapid absorption of the drug in the mucosa with subsequent passive diffusion in the lipid membrane. The literature data report that drug absorption by the sublingual and nasal routes is 3 to 10 times higher than other routes (oral and topical) [37,38,39,40]. Despite the theoretical advantages of nasal administration for the treatment of TB, our results here do not suggest significant advantages over other routes of administration. A possible explanation is that the mouse is not the ideal model for nasal administration, as the size of its nostril is small and does not allow the delivery of high viscous substances and large volumes (3 µL each nostril) into the nasal cavity. Thus, although the nasal route may be an advantageous route for drug administration for tuberculosis, we currently do not have a suitable in vivo model for testing LCSs.

## 5. Conclusions

In summary, the developed liquid crystal systems may be a viable strategy as an innovative alternative to aid in the treatment of TB. Considering that an important issue is the longevity of TB chemotherapy, often with daily oral intakes of antibiotics, the possibility of producing a slow-release nanostructured drug delivery system is encouraging. The results obtained in this manuscript showed that the use of unconventional routes of administration using a liquid crystal system for the delivery of rifampicin is plausible. The strategy proposed in this work may be a viable tool in TB therapy.

## Figures and Tables

**Table 1:** Degrees of cytotoxicity for the classification of cell samples.

Grade	Cytotoxicity	Halo Size
0	Absence	Absence of discoloration around of sample
1	Light	Discoloration zone limited to the area under the sample
2	Mild	Discoloration zone around the sample up to 0.5 cm
3	Moderate	Discoloration zone from 0.5 to 1.0 cm around the sample
4	Severe	Discoloration zone greater than 1.0 cm

**Table 2:** Selected liquid crystal systems.

System	Composition					Aspect	
	PPD (%)	Oleilamine (%)	PPD: Oleilamine 1:1 (*w*:*w*) (%)	Water (%)	Procetyl^®^ AWS (%)		
**9D**	30	_	_	20	50	STVI	L. crystal
**10D**	40	_	_	10	50	STVI	L. crystal
**9E**	_	30	_	20	50	STBV	L. crystal
**10E**	_	40	_	10	50	STBV	Precursor liquid crystal system
**9F**	_	_	30	20	50	SOVI	Precursor liquid crystal system
**10F**	_	_	40	10	50	SOBV	L. crystal

**Table 3:** Selected liquid crystal systems with rifampicin.

System	Composition					
	PPD (%)	Oleilamine (%)	PPD: Oleilamine 1:1 (*w*:*w*) (%)	Water (%)	Procetyl^®^ AWS (%)	Rifampicim (µg·mL^−1^)
**9DR**	30	_	_	20	50	0.150
**10DR**	40	_	_	10	50	0.150
**9ER**	_	30	_	20	50	0.150
**10ER**	_	40	_	10	50	0.150
**9FR**	_	_	30	20	50	0.150
**10FR**	_	_	40	10	50	0.150

**Table 4:** Flow behavior (n) and consistency index (K) of the liquid crystal systems.

System		*n*	*k*
**Liquid Crystal System**	**9D**	1.95	0.059
	**10D**	0.958	1.832
	**9E**	0.917	0.185
	**10E**	0.901	0.077
	**9F**	0.787	0.434
	**10F**	0.177	3.337
**Liquid Crystal System** **with rifampicin**	**9DR**	0.540	3.452
	**10DR**	0.382	20.824
	**9ER**	0.758	0.354
	**10ER**	0.925	0.069
	**9FR**	0.829	0.337
	**10FR**	0.697	0.264
**Liquid Crystal System** **+ SNF**	**9D**	0.145	703.144
	**10D**	0.509	83.275
	**9E**	0.415	55.683
	**10E**	0.745	26.151
	**9F**	0.486	77.446
	**10F**	0.470	129.193
**Liquid Crystal System** **with rifampicin + SNF**	**9DR**	0.229	319.585
	**10DR**	0.533	59.849
	**9ER**	0.445	58.022
	**10ER**	0.491	86.391
	**9FR**	0.119	547.658
	**10FR**	0.245	292.803

**Table 5:** Values of linear regression (r), system resistance (S), and viscoelastic exponent (*n*) for the developed liquid crystal systems.

System		R	S	*n*
**Initial System (IS)**	**9D**	0.987	164.282	0.155
	**10D**	0.982	853.439	0.116
	**9E**	0.624	14.854	0.022
	**10E**	0	9.791	8 × 10^−13^
	**9F**	0.470	117.978	0.025
	**10F**	0	330.185	1.3 × 10^−11^
**IS + Rifampicin**	**9DR**	0.954	176.103	0.155
	**10DR**	0.957	86.734	0.292
	**9ER**	0.862	12.844	0.045
	**10ER**	0	9.922	6 × 10^−3^
	**9FR**	0.801	16.532	0.053
	**10FR**	0.867	205.799	0.157
**Initial System (IS)** **+ SNF**	**9D**	0.669	5970.146	0.232
	**10D**	0.902	3050.039	0.170
	**9E**	0.841	914.508	0.113
	**10E**	0.819	910.679	0.134
	**9F**	0.743	1575.645	0.107
	**10F**	0.691	2003.491	0.059
**SI + Rifampicin** **+ SNF**	**9DR**	0.454	3468.944	0.242
	**10DR**	0.725	2468.397	0.181
	**9ER**	0.949	58.022	0.445
	**10ER**	0.517	1947.099	0.132
	**9FR**	0.091	8295.673	0.062
	**10FR**	0.662	2380.354	0.174

**Table 6:** q_max_ spreading values and lamellar and hexagonal periodicity of liquid-crystalline formulations.

System		q_max1_	q_max2_	q_max3_	d_2_/d_1_	d_3_/d_1_	Structure
**Initial System (IS)**	**9D**	13,502,052.0	27,209,302.3	40,779,753.8	2	3	Hexagonal
	**10D**	13,577,291.0	21,767,587.8	43,661,650.7	2	3	Hexagonal
	**9E**	9,913,284.3	19,887,965.9	-	2		Lamellar
	**10E**	11,524,167.8	-	-			Microemulsion
	**9F**	9,610,123.12	19,288,645.7	-	2		Lamellar
	**10F**	10,897,172.8	-	-			Microemulsion
**IS + Rifampicin**	**9DR**	13,555,631.6	27,173,962.6	41,005,472.0	2	3	Hexagonal
	**10DR**	13,578,263.8	27,088,719.0	43,661,650.7	2	3	Hexagonal
	**9ER**	10,257,637.9	20,251,938.0	-	2		Lamellar
	**10ER**	11,737,346.1	-	-			Microemulsion
	**9FR**	9,664,842.6	19,418,604.7	-	2		Lamellar
	**10FR**	10,997,492	-	-			Microemulsion

**Table 7:** Qualitative results of cytotoxicity obtained for the studied liquid crystal systems.

System	Halo (mm)	Cytotoxicity
9D	8.7	Moderate
10D	8.8	Moderate
9E	12	Moderate
10E	7.7	Moderate
9F	6.7	Moderate
10F	5.9	Moderate
9DR	5	Light
10DR	5.8	Moderate
9ER	8.5	Moderate
10ER	7.4	Moderate
9FR	6.8	Moderate
10FR	6.6	Moderate
